# Supplementary material for: Computational Study of Methane C–H Activation by Main Group and Mixed Main Group–Transition Metal Complexes
Source: Molecules. 2020 Jun 17;25(12):2794. doi: 10.3390/molecules25122794 (PMC7355694; doi:10.3390/molecules25122794)
Supplement: Supplementary file 1 [file molecules-25-02794-s001.zip › molecules-831342-supplementary.pdf]

Supporting Information for

Computational study of methane C-H activation by main group and mixed main group-transition metal complexes

by Carly C. Carter, Thomas R. Cundari*

University of North Texas, Department of Chemistry, Center of Advanced Scientific Computing and Modeling, Denton, TX 76203.

CSD refcodes from Chart 1: I (WIWCIQ), II (XOYNEG), III (LIKSEE), IV (ICOKUH), and V (ICOKIV).

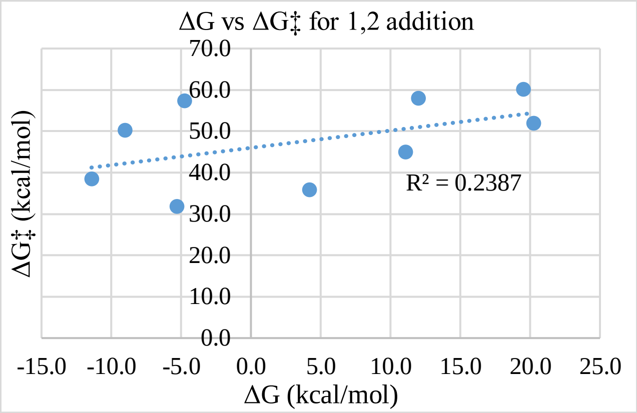

Figure S1: Plot of ΔG vs ΔG‡ for the 1,2 addition for all of the studied combinations.

Table S1: Comparisons of Functionals for BeAl molecule

Functionals	1,2 ΔG (kcal/mol)	2,1 ΔG (kcal/mol)	1,2 TS ΔG‡ (kcal/mol)
B3LYP	-5.3	-0.3	32.1
MP2	-4.7	-0.4	30.3
M06	-5.6	-2.2	27.2
wB97XD	-4.4	-0.3	29.3
Average	-5.0	-0.8	29.7
SD	0.5	2.3	1.8

Table S2: Comparisons of solvent effects (THF) for BeAl molecule with different functionals

Functional	1,2 ΔG (kcal/mol)	2,1 ΔG (kcal/mol)	1,2 TS ΔG‡ (kcal/mol)
B3LYP w/ solvent	-5.0	-3.9	33.6
MP2 w/ solvent	-5.3	-5.1	32.0
M06 w/ solvent	-5.7	-6.1	29.6
wB97XD w/ solvent	-4.3	-4.5	31.5
Average	-5.1	-4.9	31.7
SD	0.59	0.96	1.6

MgB Activating Complex

For the activating complex containing Mg and B, the products of the two reactions with bond lengths can be seen in **Figure S2** and **Figure S3**. The Gibb's free energy for the 1,2 and 2,1 addition of methane -4.2 and -7.2 kcal/mol, respectively. From this, it can be seen that the 2,1 reaction is the more thermodynamically favorable process to activate methane, though both reactions are exergonic. The energy barrier for the 1,2 addition of methane with MgB is 41.2 kcal/mol. **Figure S4** shows the computed free energy reaction diagram for the 1,2 addition of methane to the MgB compound.

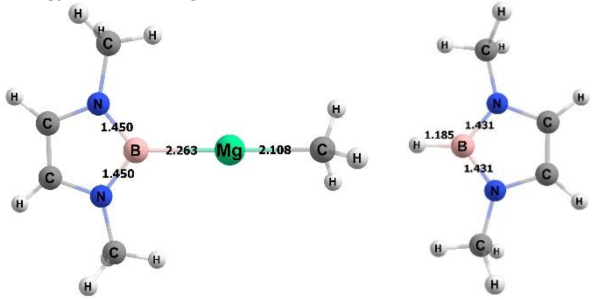

Figure S2: DFT-optimized geometries of the products of 1,2 addition of methane to the MgB complex. Bond lengths in Å.

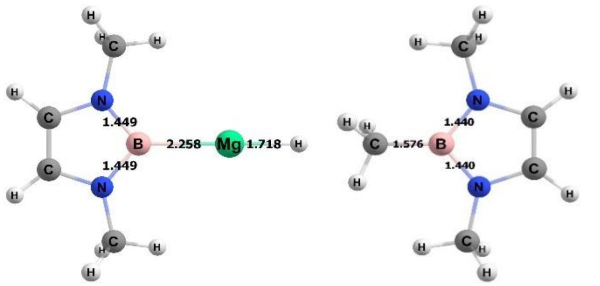

Figure S3: DFT-optimized geometries of the products of 2,1 addition of methane to the MgB complex. Bond lengths in Å.

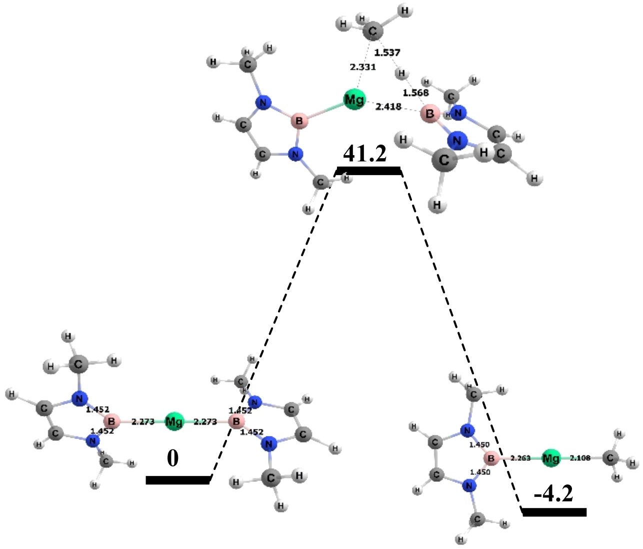

Figure S4: Reaction diagram for the 1,2 addition of methane to the MgB complex. Quoted free energies are in kcal/mol and reported relative to separated reactants. Bond lengths in Å

MgAl Activating Complex

For the activating complex containing Mg and Al, the products of the two reactions with bond lengths can be seen in **Figure S5** and **Figure S6**. The Gibb's free energy for the 1,2 and 2,1 addition of methane 11.8 and 12.3 kcal/mol, respectively. From this,

it can be seen that the 1,2 reaction is the slightly more thermodynamically favorable process to activate methane, though both reactions yield endergonic reactions. The energy barrier for the 1,2 addition of methane with MgAl is 47.2 kcal/mol. **Figure S7** shows the computed free energy reaction diagram for the 1,2 addition of methane to the MgAl compound.

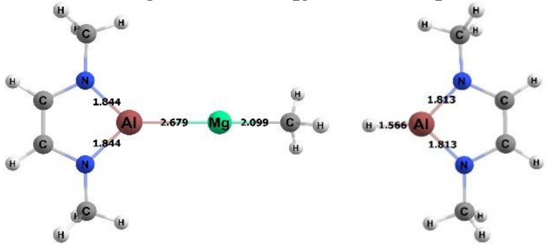

Figure S5: DFT-optimized geometries of the products of 1,2 addition of methane to the MgAl complex. Bond lengths in Å.

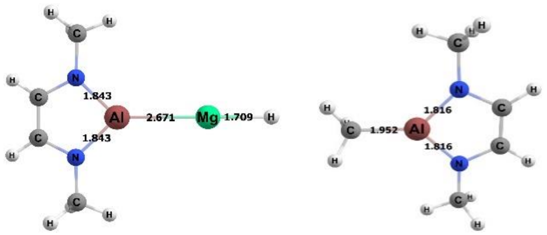

Figure S6: DFT-optimized geometries of the products of 2,1 addition of methane to the MgAl complex. Bond lengths in Å.

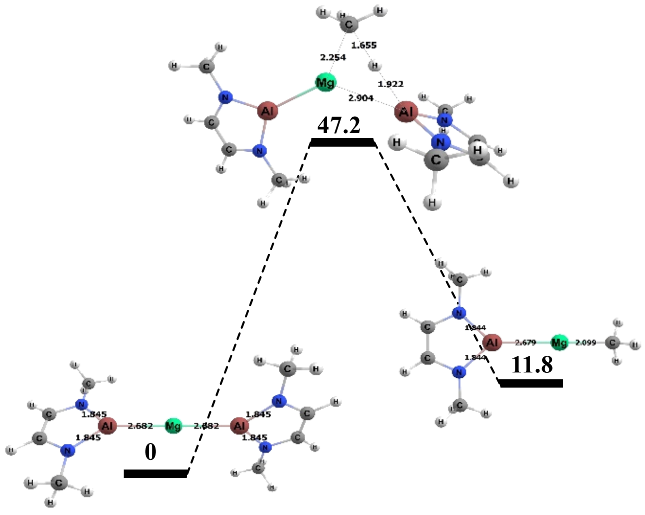

Figure S7: Reaction diagram for the 1,2 addition of methane to the MgAl complex. Quoted free energies are in kcal/mol and reported relative to separated reactants. Bond lengths in Å

MgGa Activating Complex

For the activating complex containing Mg and Ga, the products of the two reactions with bond lengths can be seen in **Figure S8** and **Figure S9.** The Gibb's free energy for the 1,2 and 2,1 addition of methane 21.1 and 22.8 kcal/mol, respectively. From this, it can be seen that the both reactions are similar in energy and are both endergonic. The energy barrier for the 1,2 addition of methane with MgGa is 48.2 kcal/mol. **Figure S10** shows the computed free energy reaction diagram for the 1,2 addition of methane to the MgGa compound.

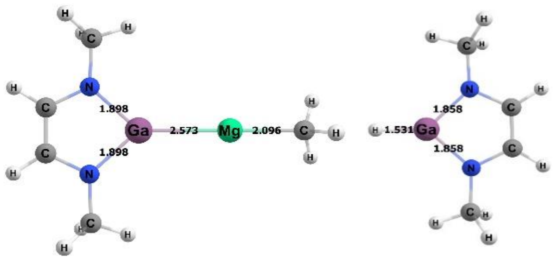

Figure S8: DFT-optimized geometries of the products of 1,2 addition of methane to the MgGa complex. Bond lengths in Å.

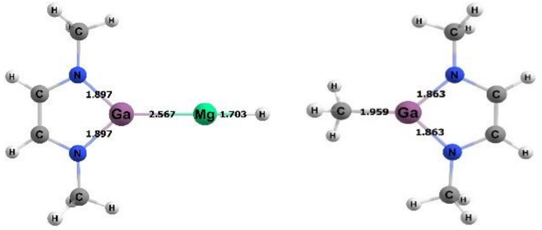

Figure S9: DFT-optimized geometries of the products of 2,1 addition of methane to the MgGa complex. Bond lengths in Å.

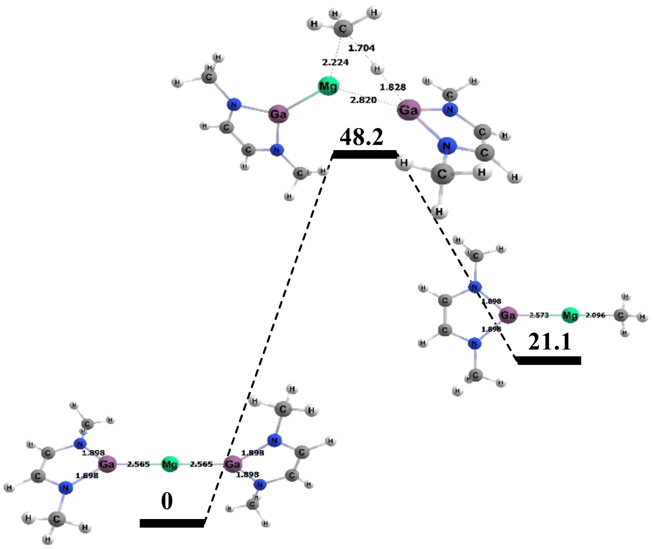

Figure S10: Reaction diagram for the 1,2 addition of methane to the MgGa complex. Quoted free energies are in kcal/mol and reported relative to separated reactants. Bond lengths in Å.

ZnB Activating Complex

For the activating complex containing Zn and B, the products of the two reactions with bond lengths can be seen in **Figure S11** and **Figure S12**. The Gibb's free energy for the 1,2 and 2,1 addition of methane -1.8 and -3.2 kcal/mol, respectively. From this, it can be seen that the both reactions are similar in energy and are both slightly exergonic. The free energy barrier for the 1,2 addition of methane with ZnB is 53.8 kcal/mol. **Figure S13** shows the computed free energy reaction diagram for the 1,2 addition of methane to the ZnB compound.

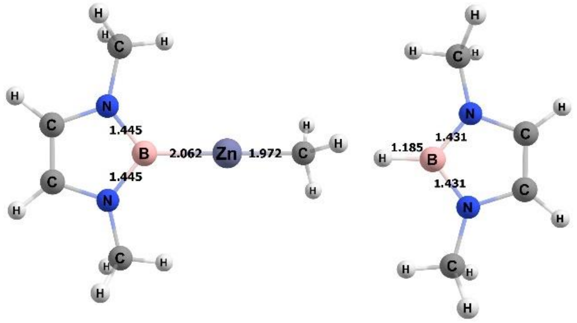

Figure S11: DFT-optimized geometries of the products of 1,2 addition of methane to the ZnB complex. Bond lengths in Å.

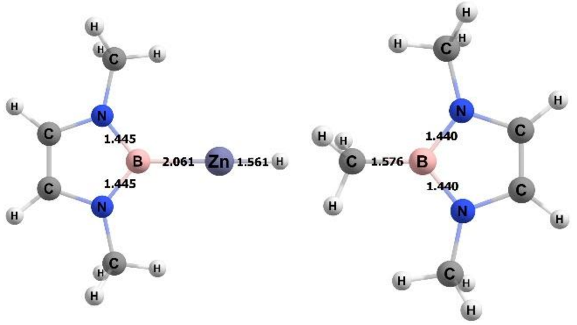

Figure S12: DFT-optimized geometries of the products of 2,1 addition of methane to the ZnB complex. Bond lengths in Å.

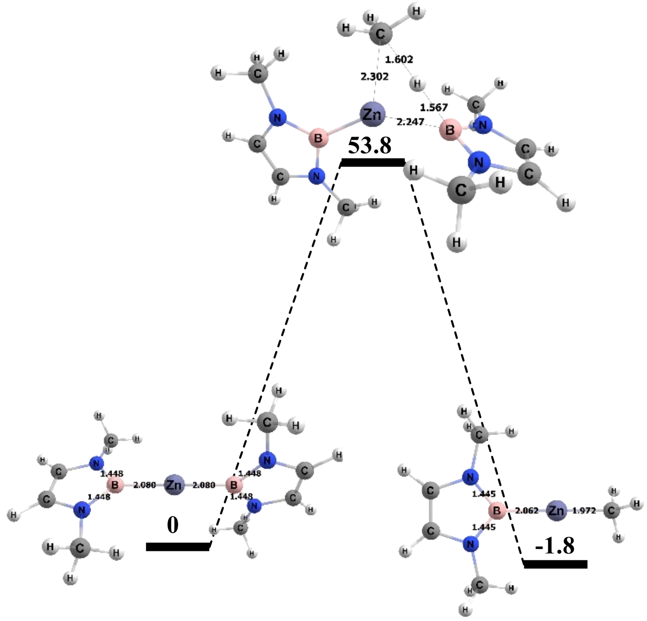

Figure S13: Reaction diagram for the 1,2 addition of methane to the ZnB complex. Quoted free energies are in kcal/mol, and reported relative to separated reactants. Bond lengths in Å.

ZnAl Activating Complex

For the activating complex containing Zn and Al, the products of the two reactions with bond lengths can be seen in **Figure S14** and **Figure S15**. The Gibb's free energy for the 1,2 and 2,1 addition of methane 12.9 and 13.1 kcal/mol, respectively. From this, it can be seen that the 1,2 addition is slightly more favorable because of its lower energy, though both methane activation reactions are endergonic. The energy barrier for the 1,2 addition of methane with ZnAl is 55.9 kcal/mol. **Figure S16** shows the computed free energy reaction diagram for the 1,2 addition of methane to the ZnAl compound.

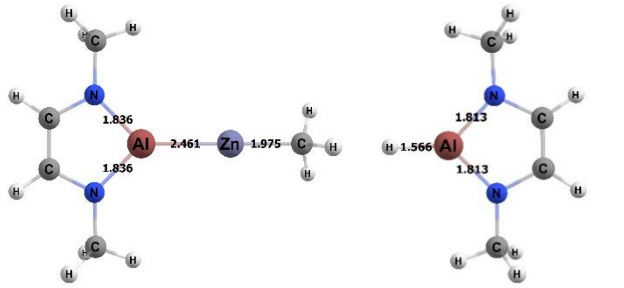

Figure S14: DFT-optimized geometries of the products of 1,2 addition of methane to the ZnAl complex. Bond lengths in Å.

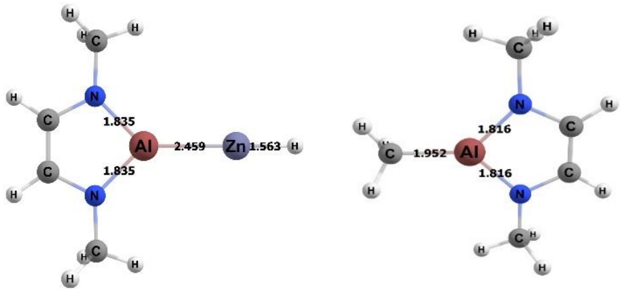

Figure S15: DFT-optimized geometries of the products of 2,1 addition of methane to the ZnAl complex. Bond lengths in Å.

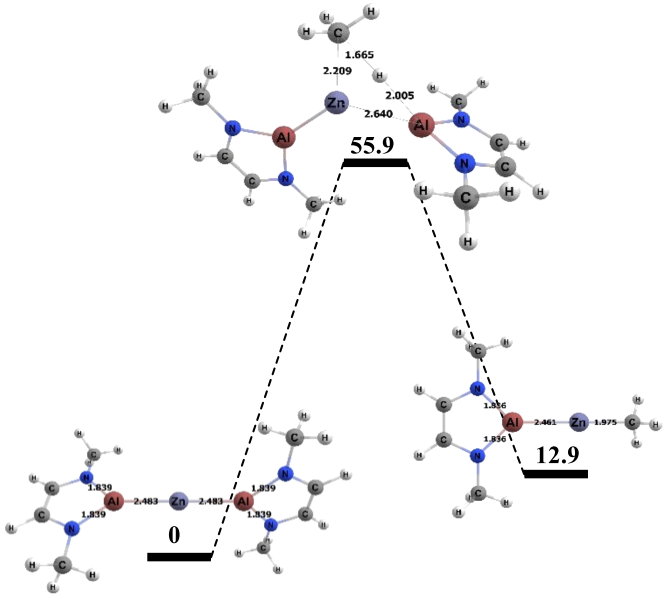

Figure S16: Reaction diagram for the 1,2 addition of methane to the ZnAl complex. Quoted free energies are in kcal/mol and reported relative to separated reactants. Bond lengths in Å.

ZnGa Activating Complex

For the activating complex containing the heaviest combination of elements investigated in this research, Zn and Ga, the products of the two methane activation reactions with optimized bond lengths can be seen in **Figure S17** and **Figure S18**. The Gibb's free energy for the 1,2 and 2,1 addition of methane 20.4 and 22.0 kcal/mol, respectively. From this, it can be seen that both reactions are very similar in energy and are both very endergonic. The energy barrier for the 1,2 addition of methane with ZnGa is 58.1 kcal/mol, which is the highest computed in this research. **Figure S19** shows the computed free energy reaction diagram for the 1,2 addition of methane to the ZnGa compound.

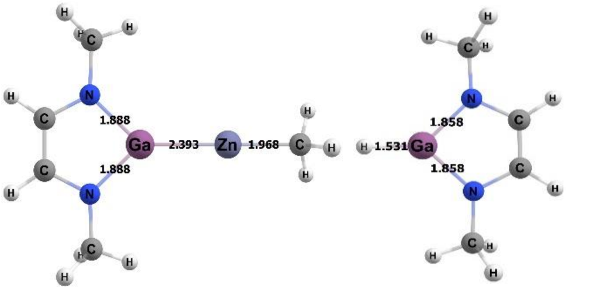

Figure S17: DFT-optimized geometries of the products of 1,2 addition of methane to the ZnGa complex. Bond lengths in Å.

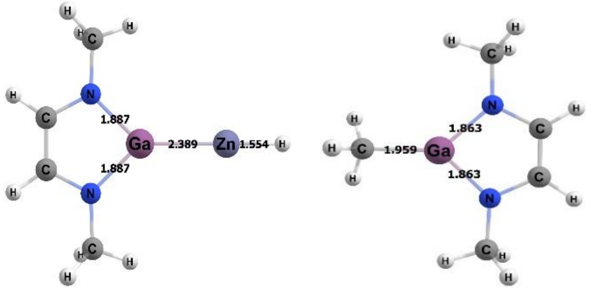

Figure S18: DFT-optimized geometries of the products of 2,1 addition of methane to the ZnGa complex. Bond lengths in Å.

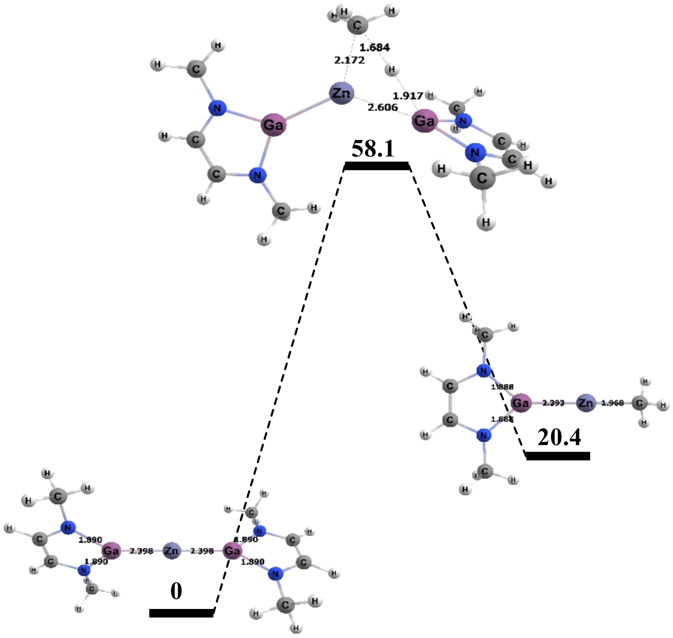

Figure S19: Reaction diagram for the 1,2 addition of methane to the ZnGa complex. Quoted free energies are in kcal/mol and reported relative to separated reactants. Bond lengths in Å.
